# Novel Cryopreservation Approach Providing Off-the-Shelf Availability of Human Multipotent Mesenchymal Stromal Cells for Clinical Applications

**DOI:** 10.1155/2019/4150690

**Published:** 2019-11-22

**Authors:** Olena Rogulska, Olga Tykhvynska, Olena Revenko, Viktor Grischuk, Svitlana Mazur, Natalia Volkova, Roman Vasyliev, Alexander Petrenko, Yuriy Petrenko

**Affiliations:** ^1^Department of Biochemistry, Institute for Problems of Cryobiology and Cryomedicine, National Academy of Sciences of Ukraine, Pereyaslavska 23, 61015 Kharkiv, Ukraine; ^2^State Institute of Genetic and Regenerative Medicine, National Academy of Medical Sciences of Ukraine, Kiev 04114, Ukraine; ^3^Department of Biomaterials and Biophysical Methods, Institute of Experimental Medicine AS CR v.v.i, Videnska 1083, 14220 Prague, Czech Republic

## Abstract

Cryopreservation is the only established method to provide long-term storage and fast availability of cellular product for therapeutic applications. The overwhelming majority of cryopreservation media contain toxic concentrations of dimethyl sulfoxide (DMSO) limiting the possibility for the direct administration of cryopreserved cells to the patients. Here, we propose a novel approach for nontoxic xeno-free cryopreservation of human multipotent mesenchymal stromal cells (MSCs) aimed at ensuring high viability, ready-to-use availability, and localized delivery of the cell-based graft into damaged tissues. For MSC cryopreservation, we applied sucrose pretreatment procedure and xeno-free cryoprotective medium containing human platelet-poor blood plasma (PPP), sucrose, and nontoxic concentration of DMSO. Using the combination of PPP, 0.2 M sucrose, and 1% DMSO, the recovery rate of cryopreserved MSCs reached 73% of the values obtained for noncryopreserved cells. Moreover, the presence of PPP in the cryoprotective medium provided the possibility to create a ready-to-use 3D hydrogel for the localized delivery and additional support of MSCs *in vivo*. In a proof-of-concept study, we assessed the regenerative capacity of cryopreserved MSCs in a full-thickness wound model in mice. The positive impact of MSCs within 3D gel on wound healing rates was confirmed by morphometric and histological examinations. Our results demonstrate the possibility to apply cryopreserved cells immediately after thawing using a cryoprotective medium as the vehicle solution.

## 1. Introduction

The unique properties of multipotent mesenchymal stromal cells (MSCs) make them an indispensable tool for regenerative medicine. It is becoming accepted that the mechanism of MSC therapeutic activity is mainly associated with their paracrine action. The secretome of MSCs comprises the number of growth factors and cytokines, as well as microvesicles and exosomes, which are involved in the transfer of proteins and miRNA to other cells [1–3]. Such interaction between MSCs and surrounding cells can prevent cell death, modulate inflammation and immune response, and provide the microenvironment necessary for normal development of resident cells [4, 5].

Although the optimal conditions of cell administration are currently unknown, there are shreds of evidence showing that the success of MSC treatment may relate to the timing of cell application. For example, the commonly applied timing for cell therapy of thermal or radiation burn wounds comprises 24 hrs–7 days, depending on the administration route [6, 7]. The fast cell delivery has been shown to be beneficial in ischemic stroke or heart disease treatment [8, 9]. However, the cell manufacturing process, which includes isolation, expansion, and quality control assessment procedures, usually lasts for several weeks or months.

The cryopreservation may represent the only possibility to provide fast availability of cellular product for therapeutic applications. The common clinical-grade cryopreservation methods include the application of 10% dimethyl sulfoxide (DMSO) in saline/electrolyte solutions, such as NaCl, Ringer's solution, or Plasmalyte®-148 [5, 10, 11]. However, high concentration of DMSO has been confirmed to be toxic towards cells at positive temperatures and its direct infusion can cause serious adverse reactions in patients [12–14]. The need for DMSO removal prior to clinical application leads to additional complications in the preparation of a final cellular product that may cause doubt on the results of prepared safety studies.

Many attempts have been made elsewhere to reduce the concentration of DMSO and develop the GMP-compliant xeno-free media for stem cell banking [15–19]. In terms of clinical application, hydroxyethyl starch- (HES-) based cryoprotective solutions (CPS) with reduced DMSO concentration represent additional interest, since 6-10% HES-containing solutions are commercially available as plasma substitutes (e.g., Volulyte 6%, Fresenius Kabi, or Tetraspan® 6%/10% from B. Braun Medical). The application of 5% DMSO/5% HES solution provided more than 70% viability of rat MSCs, human fibroblasts, or keratinocytes [20, 21]. However, even with extracellular support given by HES, the concentration of DMSO usually remains high (more than 5%) and should be reduced prior to application.

The development of cryopreservation methods providing ready-to-use availability and the possibility for direct administration of the final cellular grafts to a patient without any additional manipulations would have great advantages in a faster translation of safe MSC-based products to the clinic. These methods, in turn, should be based on the use of safe and clinically applicable compounds and techniques. Among the natural substances able to serve as potential components in CPS, human blood-derived products are of great interest, since they are highly employed in regenerative medicine and tissue engineering. Confirmed biocompatibility, high accessibility, and established regulatory policy promote the development of new products and treatment protocols using human blood plasma as a therapeutic agent. The presence of fibrin in blood plasma opens the opportunities for the generation of three-dimensional (3D) hydrogels that could serve as scaffolds for localized cell delivery in regenerative medicine and tissue engineering [22, 23].

These findings have promoted the application of human plasma in clinical stem cell banking. It has been shown that human plasma can be used as a viable alternative to fetal bovine serum- (FBS-) containing media for the cryopreservation of MSCs from human and rat bone marrow [24]. Smagur et al. have reported that human serum albumin in a cryoprotective medium may be replaced by human plasma without negative impact on recovery, clonogenic potential, and engraftment of peripheral blood hematopoietic stem/progenitor cells [25]. However, the amount of DMSO in these solutions remains high, and thus, to reduce the DMSO concentration and provide the ready-to-use availability of the MSC graft, following optimization of the approach is needed. In this regard, we have previously proposed an alternative way for the cryopreservation of MSCs based on the sugar (sucrose, trehalose, and raffinose) pretreatment prior to cryopreservation [26]. This protocol allowed us to preserve more than 50% of cells without the application of DMSO or other penetrative cryoprotectants [26] and provided the opportunities for its further optimization to obtain higher cell survival [27].

The idea of the current study was built on the hypothesis that the substitution of basal saline culture medium in CPS by human blood plasma can improve the outcomes of sugar pretreatment-based cryopreservation of MSCs and simultaneously provide the possibilities for localized delivery of cryopreserved MSC grafts within CPS in the form of 3D plasma-based hydrogel.

## 2. Materials and Methods

### 2.1. Cell Isolation and Culture

After receiving the written consent of informed healthy volunteer donors, human adipose tissue MSCs were isolated from lipoaspirate of adult patients in strict accordance with the recommendations of the World Medical Association Declaration of Helsinki. MSCs were obtained by collagenase digestion using the previously described method [27, 28]. Isolated MSCs were cultured in T25 adhesive polystyrene cell culture flasks (TPP, Switzerland) at 37°C, 5% CO_2_, and 95% humidity in Minimal Essential Medium-*α* modification (*α*-MEM, Sigma-Aldrich, USA) containing 10% FBS (Biowest, France), 50 *μ*g/ml penicillin (Biowest, France), 50 *μ*g/ml streptomycin (Biowest, France), and 0.2 mM L-glutamine (Sigma-Aldrich, USA). Complete medium changes were performed every 3–4 days. On reaching 80% confluence, the cells were trypsinized, counted with a hemocytometer, and subcultured for 4 passages. *In vitro* expanded MSCs at passage 4 were harvested, characterized as previously described [27], and used in further experiments.

### 2.2. Blood Plasma Preparation

To avoid the impact of platelets and platelet-derived factors on the properties of MSCs during *in vitro* culture or implantation *in vivo*, we have chosen platelet-poor plasma (PPP) as a less bioactive CPS component. The whole blood units of 5 different volunteer adult donors were collected at Kharkov Regional Blood Service Center accredited by the Ministry of Health of Ukraine. Before the collection, all donors were tested according to current Ukrainian legislative guidelines. One part of the blood (30 ml) was collected in the presence of an anticoagulant and processed by two-stage centrifugation to separate PPP (<5 · 104 platelets/*μ*l) from platelet-rich plasma (PRP) fraction [29, 30]. Additionally, another 10 ml of blood was collected without an anticoagulant and stored at room temperature during 30 min for spontaneous coagulation. The obtained blood serum was aliquoted and stored at -20°C until further use.

### 2.3. Cell Pretreatment and Cryopreservation

MSCs were subjected to the pretreatment procedure, which comprised cell culture in complete medium, supplemented with 0.1 M sucrose for 24 hrs prior to cryopreservation [26, 27]. Pretreated cells were trypsinized and resuspended in 1 ml CPS. CPS was composed of either *α*-MEM or PPP, supplemented with 0.2 M sucrose and DMSO in different concentrations (0, 1, 2, 5, and 10%). After a 5 min incubation in corresponding CPS at 2-10°C, 300 *μ*l of a cell suspension (1.8 × 106 cells) was placed into 1 ml cryovial (NUNC, USA) and cryopreserved with the cooling rate of 1 degree/min down to -80°C, using Nalgene® Mr. Frosty freezing container followed by immersion into liquid nitrogen. Cryovials were stored in liquid nitrogen for one month and thawed in a water bath at 37°C before further studies.

### 2.4. Assessment of Postthaw Cell Viability and Recovery

Immediately after thawing, cell viability was tested using a Trypan blue plasma membrane integrity assay according to the standard protocol. Viability rate was expressed as a percentage of viable unstained cells in suspension related to the total number of cells counted in a hemocytometer (*n* = 8).

To test cell recovery, cryopreserved MSCs were seeded into standard 96-well culture plates (TPP, Switzerland) in a concentration of 5 × 103 cells/cm^2^ and cultured for 24 hrs. After 24 hrs, the Alamar Blue test (AB, Serotec Ltd, USA) was performed as described previously [27]. Briefly, cells were incubated in the complete culture medium, supplemented with 10% AB during 3 hrs. Afterwards, the fluorescence level of the AB solution was assessed using a TECAN GENios microplate reader (Tecan Genios, Austria) with an excitation wavelength of 550 nm and an emission wavelength of 590 nm. The ratio of the fluorescence intensity of experimental and blank sample (without cells) was used as AB value and expressed in relative fluorescence units (RFU) (*n* = 8). Noncryopreserved cells served as a control group.

### 2.5. Preparation of 3D PPP-Based Hydrogel

A PPP fraction was mixed with 10% calcium chloride solution and blood serum in a ratio 9 : 0.25 : 0.75. To obtain MSCs embedded in hydrogel, the PPP fraction was preliminarily supplemented with a cell suspension in a concentration of 5 · 106 cells per 1 ml of the final mixture. In several experiments, the PPP fraction was supplemented with 0.2 M sucrose and 1% DMSO (PS1D solution).

### 2.6. Morphology, Viability, and Metabolic Activity of MSCs within PPP-Based Gel

Morphology and viability of MSCs within PPP-based 3D gels were estimated before cryopreservation, immediately after thawing, and following 24 hrs, 48 hrs, and 120 hrs of postthaw recultivation. Cell morphology was assessed using a double fluorescent staining with fluorescein diacetate (FDA) and ethidium bromide (EB) [31]. FDA/EB staining was analysed with Zeiss LSM 510 META (Carl Zeiss, Germany). Confocal images were obtained along the *z*-axis with 20 *μ*m intervals at an excitation wavelength of 488 nm for FDA and 543 nm for EB.

The metabolic activity of MSCs within the PPP-based gels was assessed by the AB assay. However, since the gel decelerated AB diffusion, the incubation time was increased from 3 hrs to 24 hrs. The fluorescence level of AB solution was measured by a TECAN GENios microplate reader (Tecan Genios, Austria) as described above. The proliferation of MSCs within PPP-based gels was assessed by the AB assay in the same samples after 3D cell culture during 1, 3, and 5 days. Each experiment was repeated in triplicate.

### 2.7. Experimental Wound Model

Adult male Balb/C mice (*n* = 27) weighing 25–30 g at the age of 5-6 months were used for modelling of full-thickness excisional skin wounds. Animals were kept under standard conditions with free access to food and water. Mice were housed and treated in strict accordance with the guidelines for the care and use of laboratory animals of the “4th European Convention for the Protection of Vertebrate Animals” (ETS 123, Strasbourg, France, 1986). The experimental protocol was approved by the Local Institute Committee on Ethics and Welfare of the Institute for Problems of Cryobiology and Cryomedicine.

All surgical interventions in animals including decapitation were performed under anaesthesia induced by a mixture of 2% Sedazine (Biovet, Poland) and 1% propofol (Cleric Life Sсiences Limited, India) in the doses of 0.1 ml/100 g body weight and 0.2 ml/100 g body weight, respectively.

Before the surgery, animals were anaesthetized; their dorsal skin was shaved and then disinfected with 10% povidone-iodine. Full-thickness skin wounds were formed in compliance with the rules of aseptic and antiseptic techniques using a steel biopsy punch (6 mm diameter, “Stiefel,” Germany) [32]. To prevent contraction, the edges of the wounds were fixed with a polymer medical plaster (“Dr. House,” China) and glue BF-6 (“Lubnyfarm,” Ukraine). Each mouse had one wound on the left side and one wound on the right side of the back (e.g., 54 wounds in the study).

All wounds (*n* = 54) were blindly divided into 4 groups: group 1 (*n* = 13)—control, spontaneous healing; group 2 (*n* = 13)—3D gel containing PPP, 0.2 M sucrose, and 1% DMSO; group 3 (*n* = 14)—3D gel containing PPP, 0.2 M sucrose, 1% DMSO, and noncryopreserved MSCs (0.25‐0.3 × 106 cells in 50 *μ*l); and group 4 (*n* = 14)—3D gel containing MSCs (0.25‐0.3 × 106 cells in 50 *μ*l) cryopreserved in a solution composed of PPP, 0.2 M sucrose, and 1% DMSO.

Wound surfaces were covered with a semipermeable polyurethane membrane (Tegaderm, Germany); elastic bandage (Coban, Germany) was used as the outer dressing. Each animal was then housed alone in a separate cage to avoid any further wound damage.

### 2.8. Evaluation of Wound Healing

Skin wounds were examined on postwounding days 3, 7, 14, and 28. Animals were sedated and individual digital images were taken. The images were then analysed using the ImageJ 1.50b software to assess the wound surface area. The percentage of the wound closure was estimated by the formula

(1) $$\begin{array}{c} \frac{S_{0}-S_{t}}{S_{0}}\times 100\%, \end{array}$$

where *S*_0_ is the initial wound area; *S*_*t*_ is the wound area at day *t*.

For histological examination, cutaneous biopsy specimens were excised, fixed in a 10% solution of buffered formalin, and embedded in TissueTec (“O.C.T. p.,” UK). The 5-6 *μ*m serial sections were obtained on the cryotome Slee Cryostat MEV (“Slee Medical” GMBH, Germany). After standard dehydration, sections were stained with hematoxylin/eosin (H&E) in accordance with standard procedures and covered with Canadian balsam. The obtained histological preparations were studied using the microscope CETI EpiFluor (CETI, Seraing, Belgium).

### 2.9. Assessment of Vascularization

The number of capillary vessels, in a standardized field of a histological section stained with H&E, was counted using light microscopy and image analysis software ImageJ 1.50b. Assessments were performed in blinded specimens examined in random order. In groups of the 3rd postwounding day, adjacent to wound area tissue was analysed. The mean number of vessels within the 3 areas (magnification 200x) of each histological section (*N* = 5) was calculated and used for statistical analysis.

### 2.10. Detection of Growth Factors in Conditioned Medium

For conditioned medium preparation, 1 × 106 MSCs (*N* = 6) were seeded either in T25 culture flasks (TPP, Switzerland) or embedded into the 3D PPP-based hydrogels. After 24 hrs of culture in *α*-MEM (Sigma-Aldrich, USA) containing 10% FBS (Biowest, France), the medium was changed to serum-free *α*-MEM and cells were cultured for another 24 hrs. The concentration of the Fibroblast Growth Factor 2 (FGF-2) and Vascular Endothelial Growth Factor (VEGF) in a conditioned medium was determined by the multiplex analysis using the Bio-Plex™ custom-made 27-plex kit (Bio-Rad, USA) according to manufacturer instructions. The data was obtained using the automatic photometer for Bio-Plex microplates (Bio-Plex® 200 Systems, Bio-Rad, USA) and the Bio-Plex Manager (“Bio-Rad”) software. The FGF-2 and VEGF concentrations were determined from the calibration curve for each growth factor (the dynamic range 0.22–32000 pg/ml) according to the manufacturer recommendations.

### 2.11. Statistical Analysis

Data was expressed as mean ± SD with *n* indicating the number of independent experiments. To quantify differences between multiple groups, the Kruskal-Wallis test was used with *p* ≤ 0.05 considered as significant. In the studies on growth factor detection in a conditioned medium, the paired *t*-test was applied. Obtained results were processed using the Past v. 3.0 statistical software package.

## 3. Results

### 3.1. Effect of CPS Composition on Viability and Recovery of MSCs after Cryopreservation

Prior to cryopreservation, all samples had a viability of 96.2 ± 3.6%. In line with our previous work [26], combined sucrose application as a component of CPS and pretreatment supplement during cell culture led to a significant improvement of the postthaw cell viability (up to 47.3 ± 5.9%) obtained in the absence of DMSO or PPP.

**Figure 1:**
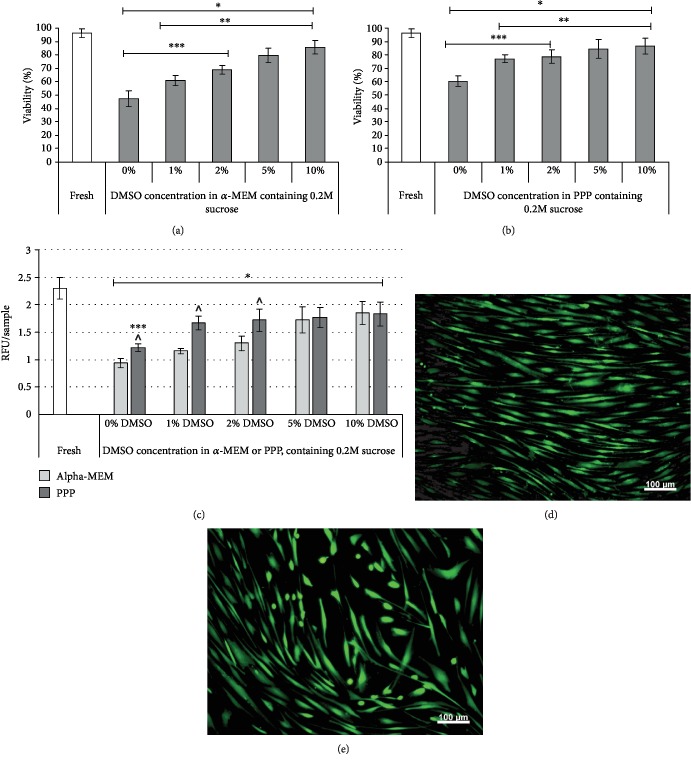
Effect of CPS composition on postthaw MSC viability and recovery. (a) Cell viability after cryopreservation in *α*-MEM containing 0.2 M sucrose and 0-10% DMSO. (b) Cell viability after cryopreservation in PPP containing 0.2 M sucrose and 0-10% DMSO. (c) Cell recovery assessed by Alamar Blue test after cryopreservation in *α*-MEM or PPP containing 0.2 M sucrose and 0-10% DMSO. (d) Viability (FDA/EB staining) and morphology of MSCs before cryopreservation; (e) viability (FDA/EB staining) and morphology of MSCs after cryopreservation using PPP/0.2 M sucrose/1% DMSO mixture (PS1D) and subsequent 24 hrs culture. ^∗^Data is significantly different (*p* < 0.05) compared to fresh control; ^∗∗^data is significantly different (*p* < 0.05) compared to 0% DMSO; ^∗∗∗^data is significantly different (*p* < 0.05) compared to 10% DMSO; ^data is significantly different (*p* < 0.05) compared to *α*-MEM group with the same DMSO concentration. Statistical analysis was performed by the Kruskal-Wallis test.

In the next set of experiments, pretreated MSCs were cryopreserved in *α*-MEM or PPP using 0.2 M sucrose and varying (1–10%) concentrations of DMSO (Figure 1(a)). The viability of MSCs enhanced with an increase of DMSO concentration. In the 1% DMSO/99% *α*-MEM group, the cell viability rate comprised 60.8 ± 3.9%. When DMSO concentration was raised to 2%, 5%, and 10%, the MSC viability increased to 68.9 ± 4.2%, 79.6 ± 5.3%, and 85.6 ± 4.1%, respectively.

As shown in Figure 1(b), the replacement of *α*-MEM by PPP led to the significant improvement of the MSC viability using only 0.2 M sucrose as a cryoprotectant (60.4 ± 3.9%). The addition of 1% DMSO to a PPP-based CPS significantly enhanced cell viability to 77 ± 2.9%. The further increase of DMSO concentration to 2%, 5%, and 10% led to an additional enhancement of MSC viability, reaching maximum at 10% DMSO (86.4 ± 5.9%).

Considering that cryopreservation may trigger apoptosis, the accurate assessment of MSC preservation requires the evaluation of cell survival parameters after additional postthaw cell recultivation. In the current study, we examined MSC recovery 24 hrs after thawing using the AB assay (Figure 1(c)).

The recovery of MSCs cryopreserved in *α*-MEM containing 0.2 M sucrose and different concentrations of DMSO increased depending on the applied amount of DMSO (Figure 1(c)). When *α*-MEM was substituted by PPP, cryopreserved cells showed higher recovery rate 24 hrs after thawing. This beneficial effect was especially prominent in CPS with 0%, 1%, or 2% of DMSO. The supplementation of PPP/0.2 sucrose CPS with 1% DMSO resulted in a significant 27% increase of MSC recovery (1.67 ± 0.13 RFU). The following increase of DMSO concentration within CPS did not additionally enhance the MSC recovery, providing similar to 1% DMSO outcomes, which corresponded to 73% of the values obtained for noncryopreserved cells (Figure 1(c)). There was no significant difference between this group and the recovery of MSCs cryopreserved using the mixture of *α*-MEM, 0.2 M sucrose, and 10% DMSO (*p* = 0.1553).

The MSC morphology after cryopreservation in such conditions (Figure 1(e)) was not visibly different compared to noncryopreserved cells (Figure 1(d)).

Considering these results and previously reported DMSO potential toxicity, in further studies we applied developed PPP/sucrose-based CPS, supplemented with 1% DMSO (PS1D), as the least toxic composition providing effective protection of MSCs during cryopreservation.

### 3.2. Properties of MSCs Cultured in PS1D Gel

In the next series of experiments, we assessed whether PS1D (PPP/sucrose/1% DMSO mixture) could serve as a vehicle solution for the localized MSC delivery into the damaged area. Following calcium and serum addition, MSC-containing PS1D solution polymerized and formed stable hydrogel constructs. During *in vitro* culture, cells were able to spread and form the 3D cellular network, confirming the suitability of using PS1D solution for localized cell delivery.

**Figure 2:**
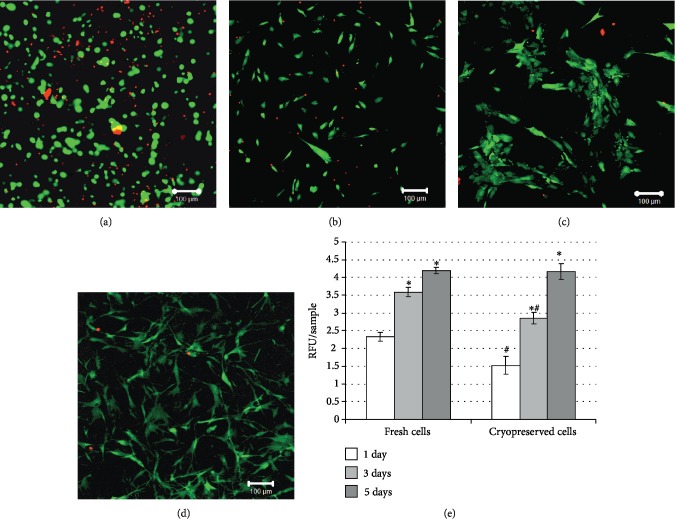
Morphology and viability (FDA/EB staining) of MSCs cryopreserved in PS1D (PPP, containing 0.2 M sucrose and 1% DMSO) and cultured as 3D hydrogel. For the hydrogel formation, samples were mixed with 10% calcium chloride solution and blood serum of the same donor in ratio 9 : 0.25 : 0.75. Cell viability within 3D hydrogel (a) 24 hrs, (b) 48 hrs, (c) 72 hrs, and (d) 120 hrs after thawing. Proliferation rate of MSCs during culture in PS1D-based gel assessed by Alamar Blue test (e). ^∗^Data is significantly different (*p* < 0.05) compared to the 1^st^ day of culture; ^#^data is significantly different (*p* < 0.05) compared to fresh cells. Statistical analysis was performed by the Kruskal-Wallis test.

After cryopreservation of MSCs in PS1D and following 3D gel formation, the cell spreading was delayed compared to noncryopreserved samples. After 24 hrs of postthaw 3D cell culture, MSCs in PS1D-based gel remained round (Figure 2(a)). However, following 48 hrs of culture, MSCs attained their specific shape (Figure 2(b)), and after 72 hrs, the majority of cells were viable and had fibroblastic morphology (Figure 2(c)). On day 5 of culture, cryopreserved MSCs proliferated and formed the 3D cell network (Figure 2(d)).

As shown in Figure 2(e), the metabolic activity of cells in PS1D-based gel immediately after cryopreservation was lower compared to that of fresh MSCs. However, on the 3^rd^ day of culture, the values of cryopreserved cells increased and reached 79% of fresh control, confirming cell proliferation. After 5 days of culture, no significant differences between cryopreserved and noncryopreserved MSCs were detected (Figure 2(e)).

### 3.3. Cryopreserved MSCs in PS1D Gel Promoted Wound Closure and Improved Skin Repair

To examine the potential benefits of PS1D as a system for the delivery of cryopreserved MSCs to a damaged area, we applied a full-thickness skin excision wound model in mice.

**Figure 3:**
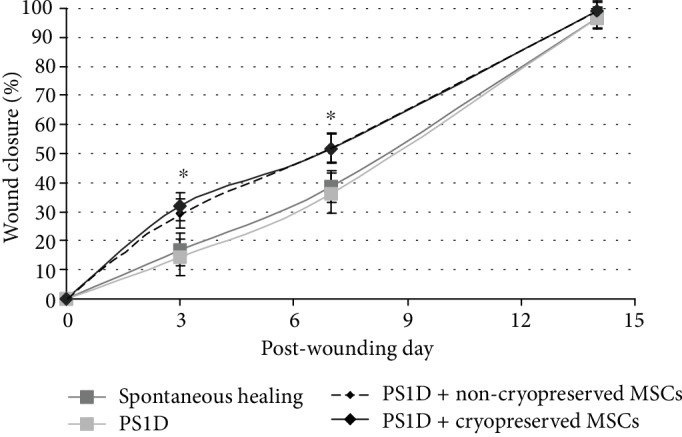
The healing rates of full-thickness skin excision wounds in mice after application of fresh or cryopreserved MSCs within PS1D-based hydrogel (PPP, containing 0.2 M sucrose and 1% DMSO). PS1D group represents the cell-free hydrogel. PS1D+noncryopreserved MSC group represent freshly expanded cells, resuspended in PS1D solution. PS1D+cryopreserved MSC group represents MSCs cryopreserved by the proposed protocol, using PS1D as the cryoprotective solution. Prior to application on the full-thickness skin excision wounds in mice, samples were mixed with 10% calcium chloride solution and blood serum of the same donor in ratio 9 : 0.25 : 0.75. ^∗^Data is significantly different (*p* < 0.05) compared to spontaneous healing and PS1D alone group (Kruskal-Wallis test).

Wound healing was evaluated throughout the 2-week period by visual inspection and planimetric analysis. The initial wound diameter in all groups was 6 mm (Fig. [Supplementary-material supplementary-material-1]). Wound sizes in the control group (spontaneous healing) and the cell-free PS1D group were comparable during the entire observation period (Figure 3). The percentages of wound closure were 16.9 ± 6.5% and 14.3 ± 6.3% at day 3 and 38.5 ± 5.5% and 36.3 ± 7.1% at day 7, respectively (Figure 3, Fig. [Supplementary-material supplementary-material-1]).

When wounds were treated by MSCs using 3D PS1D-based hydrogel as a vehicle solution, the process of epithelial overgrowth was faster compared to spontaneous healing and PS1D alone group. MSCs in PS1D-based gel provided 2 times faster wound closure at day 3, and this stimulating effect persisted over a week after application. No significant differences in the rates of wound closure were observed between cryopreserved and noncryopreserved MSCs applied within PS1D hydrogel. By the 14^th^ day of the experiment, wounds in all studied groups were completely epithelized.

**Figure 4:**
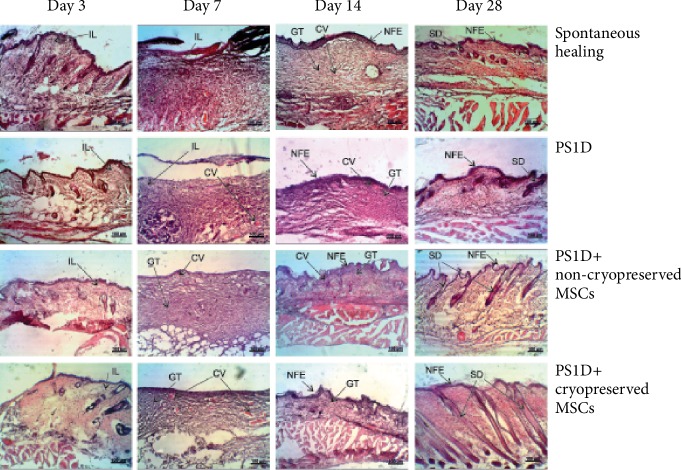
Histological sections of excision wounds at days 3, 7, 14, and 28 of the healing process (H&E staining). CV: capillary vessels; IL: infiltration by leukocytes; GT: granulation tissue; NFE: newly formed epithelium; SD: skin derivative. Full-thickness skin excision wound model in mice. The figure shows representative images of the histological sections within each corresponding group. No significant variations in the treatment response within each group were observed.

To assess the regeneration of skin epidermal and dermal layers, we examined the histological sections of cutaneous wounds at different time points (Figure 4). The results of histological analysis were similar among the animals with minimal variations in treatment response within each group.

Histological analysis of skin sections at day 3 revealed that necrotic wound sites were infiltrated with polymorphonuclear leukocytes in the spontaneous healing group and the PS1D alone group (Figure 4).

After the MSC application (both cryopreserved and noncryopreserved), the inflammation was less pronounced, and we observed the first signs of granulation tissue formation.

On the 7^th^ postwounding day, after PS1D application the total number of leukocytes decreased compared to spontaneous healing. Treatment with nonfrozen and cryopreserved cells led to the formation of more matured granulation tissue than in the cell-free groups 1 and 2. Newly formed capillary blood vessels and connective tissue cells of various shapes and sizes were observed in the damaged area of all studied groups. Application of noncryopreserved as well as cryopreserved MSCs enhanced the vascularization process (Table 1).

By the end of the second week, we noticed the complete epithelialization of the wound defects in all groups. H&E staining of skin sections at this stage revealed a large number of collagen-producing fibroblasts. In addition, when treated with MSCs in PS1D gel, we observed the formation of skin derivatives. The number of capillary vessels in all groups did not change compared to day 7 of examination.

Finally, by day 28, the defect sites in the control group were filled with dense scar tissue composed of coarse bundles of collagen fibers. The newly formed full-layer epidermis had a smoothened microrelief and increased thickness compared to unimpaired skin. In contrast, collagen fibers in the PS1D group were orderly oriented and the epidermis had a normal structure. The average number of capillary vessels decreased in PS1D-based groups that served as an indication of healing process completion. Application of fresh and cryopreserved MSCs not only accelerated maturation of granulation tissue but also resulted in complete repair of skin epidermal and dermal layers.

## 4. Discussion

The current study shows that the cryoprotective solution composed of human blood plasma, supplemented with 0.2 M sucrose and 1% DMSO, ensures high viability and recovery of MSCs after sugar pretreatment-based cryopreservation and provides the possibility for localized delivery of cryopreserved MSC grafts within a 3D plasma-based hydrogel.

The “ready-to-use” concept is a challenging task in clinical-grade cryobanking of cells. The possibility to apply cellular product immediately after thawing, using CPS as a vehicle solution, may significantly simplify and fasten cell administration procedure. Since the cell-containing vial is opened just before application and used without any additional manipulations (e.g., DMSO removal), there is no need for repeating the bacteriological quality control testing or preparing the preexpansion procedure. The selection of the system and route of cell administration, for example, injection of cell suspension in a liquid form or implantation of cells within the scaffold or hydrogel, is another important issue. Thus, the development of vehicle solution, which may provide flexibility of choice, would bring much impact in translational stem cell research.

We hypothesized that the introduction of human blood plasma into CPS can allow us to solve several tasks:

- Increase the efficiency of MSC cryopreservation using sugar pretreatment approach
- Provide the flexibility in choosing the ways for further application of cryopreserved MSCs (either in conventional liquid form or as 3D hydrogel system)

To avoid the impact of platelets and platelet-derived factors on the MSC properties during *in vitro* culture or implantation *in vivo*, we have chosen PPP as a less bioactive CPS component. It allowed us to focus on the MSC survival and therapeutic activity and properly evaluate the obtained results (especially during animal studies).

We applied the sucrose pretreatment procedure, which allows achieving more than 50% cell recovery without the application of DMSO and preserving the specific immunophenotype of MSCs and their multilineage differentiation capacity [26, 27]. Sucrose is one of the most frequently used disaccharides in cryobiology studies. As an extracellular cryoprotectant, it increases the osmolarity of the medium and reduces the formation of ice crystals in the interior of the cell during the freezing process, providing stabilization of the plasma membrane [33, 34]. Sucrose has been successfully applied to reduce the concentration of DMSO to 5% during cryopreservation of umbilical cord blood stem cells [33] or replace the fetal bovine serum during cryopreservation of umbilical cord tissue [35]. Recently, benefits of sucrose application have been demonstrated for cryopreservation of 3D bioscaffolds for future clinical use [36].

We substituted saline culture medium in CPS by PPP that significantly increased the recovery of MSCs after cryopreservation without permeable DMSO. The further addition of only 1% DMSO in the CPS allowed us to obtain high (73%) recovery, which was not significantly different from 10% DMSO. That gives us a basis to propose the applied approach as a method providing ready-to-use availability of MSCs. Previously, Wang et al. have shown that the application of 1% DMSO in human plasma without sugar pretreatment provided high cell recovery rate (64 ± 2%); however, the analysis of the cellular membrane integrity, demonstrated in the study, was carried out after postthaw cell centrifugation, which usually results in the removal of dead cells and cellular debris and may provide apparent viability increase. In our study, the elimination of the sucrose pretreatment procedure resulted in only 42 ± 3% cell viability and this parameter was assessed immediately after thawing without washing procedure (data not shown).

The possibility to choose the way of further application of cryopreserved MSCs can be considered as another advantage of the proposed PS1D vehicle solution. Immediate seeding of cryopreserved MSCs within PS1D into cell culture flasks (without additional manipulations) resulted in normal attachment and spreading of cells (Figure 1(e)), confirming the possibility to apply the frozen-thawed MSCs in a conventional liquid form. Alternatively, by mixing cell-containing PS1D with pharmaceutical grade calcium solution and blood serum, both before and after cell cryopreservation, we obtained a stable 3D hydrogel immobilizing MSCs (Figure 2). We applied this feature in our proof-of-concept studies to assess the effect of cryopreservation on the MSC behaviour *in vivo* using the full-thickness wound model in mice. In that case, the PS1D-based gel was formed *in situ*. The overall study design and achieved results are summarized in Supplementary figure (Fig. [Supplementary-material supplementary-material-1]). We observed a pronounced positive impact of MSC delivery in 3D PS1D-based hydrogel on the wound healing rates (Figure 3). The wound healing capacity was similar between cryopreserved and noncryopreserved MSCs, confirming the preservation of their therapeutic properties after cryogenic storage.

**Figure 5:**
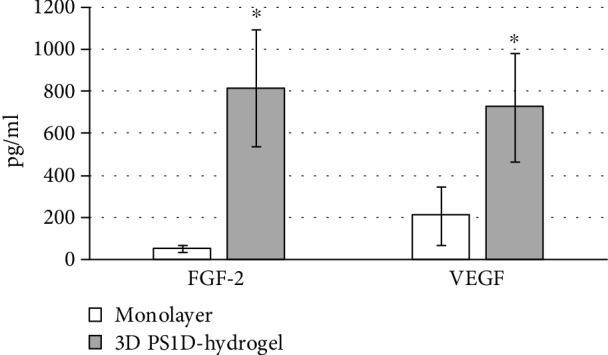
The secretion of FGF-2 and VEGF by noncryopreserved MSCs during 24 hrs of culture in the monolayer and 3D PS1D-based hydrogel. ^∗^Data is significantly different (*p* < 0.05) compared to monolayer cultures (paired *t*-test).

We believe that PS1D provides supportive conditions for MSCs within the wound microenvironment. Plasma-based hydrogels have been previously found to create optimal conditions for cell attachment, proliferation, and differentiation and act as a supportive extracellular matrix [23, 37]. Here, we showed that PS1D-based hydrogel supports growth and 3D cellular network formation of fresh and cryopreserved MSCs. Houdek et al. have reported that plasma-based hydrogels in the wound area were able to improve recruitment, growth, and differentiation of dermal-derived stem cells, leading to hair growth and sweat gland formation [37]. Combined application of plasma hydrogel and *in vitro* expanded MSCs for treatment of burns and chronic wounds promoted the restoration of the normal skin structure and accelerated the process of defect replenishment [38–40]. To confirm that the PS1D-based hydrogel system supports the paracrine activity of MSCs, we studied the levels of FGF-2 and VEGF in the conditioned medium of noncryopreserved MSCs after 24 hrs cell culture in 2D or 3D PS1D-based hydrogel conditions. As a result, the significant increase in FGF-2 (*p* = 0.03, paired *t*-test) and VEGF (*p* = 0.009, paired *t*-test) secretion was detected for PS1D-based hydrogel entrapped cells compared to monolayer cultures (Figure 5).

Both of these angiogenic factors have been previously shown to support the wound healing process (thoroughly reviewed in [41, 42]). It should be mentioned that the levels of growth factors in medium conditioned during 24 hrs by cell-free PS1D hydrogel comprised 2.3 ± 0.7 and 1.7 ± 0.4 pg/ml, respectively, for FGF-2 and VEGF.

Although we employed the xenogeneic model in the study, we did not observe any signs of acute transplant rejection during histology examination of the surrounding area of wounds. It is known that one of the main features of MSCs is their high immunomodulatory potential. Moreover, the immune privilege status of MSCs has been shown to be preserved in the xenogeneic system. It has been previously reported that the xenotransplantation of human adipose-derived stem cells into different animal species with different pathologies resulted in significant improvements independently of target disease [43]. Therefore, we can speculate that the improved wound healing rates were associated with the therapeutic action of gel-embedded MSCs. To confirm the cell-associated nature of wound healing and eliminate the immune response to xenotransplantation, we performed additional proof-of-concept experiment, using the GFP+ murine adipose tissue MSCs (supplementary [Supplementary-material supplementary-material-1]). After application of cryopreserved MSCs within the proposed hydrogel system on the full-thickness skin wounds, we observed the retention of GFP+ MSCs for up to 5 days. Herein, the wound healing rate using allogeneic cells was similar to the previously detected rate for human MSCs (data not shown). We suppose the therapeutic activity of implanted MSCs is likely connected with their initial paracrine action because engrafted cells were not detected within the wounds after 5 days of application. Presented results confirm our hypothesis and show the promise of using human blood plasma-based products in clinical-grade MSC cryopreservation and cryobanking. We propose a novel approach for nontoxic xeno-free cryopreservation of MSCs, which assures high viability, ready-to-use availability, and easy way of choosing the method for cell delivery into damaged tissues.

## Figures and Tables

**Table 1:** Vascularization of full-thickness wounds after application of PS1D-based gel alone or in combination with fresh or cryopreserved MSCs (average number of capillary vessels on histological sections).

Group/postwounding day	Day 3	Day 7	Day 14	Day 28
Spontaneous healing	3.2 ± 0.6	5.7 ± 0.6	6.1 ± 0.7	7.6 ± 0.5
PS1D	2.3 ± 0.4	9.0 ± 0.9∗	9.5 ± 0.8∗	7.0 ± 0.6
PS1D+noncryopreserved MSCs	2.2 ± 0.5	11.2 ± 1.0∗^	13.6 ± 1.2∗^	10.6 ± 0.9∗^
PS1D+cryopreserved MSCs	2.6 ± 0.3	11.9 ± 1.4∗^	13.0 ± 1.2∗^	11.1 ± 0.8∗^

^∗^Data is significantly different (*p* < 0.05) compared to the spontaneous healing group; ^data is significantly different (*p* < 0.05) compared to the PS1D group. Statistical analysis was performed by the Kruskal-Wallis test.

## Data Availability

The data used to support the findings of this study are available from the corresponding author upon request.

## Supplementary Materials

Supplementary Materials Figure S1: macroscopic images of wound closure. Figure S2: overall study design and achieved results. Supplementary file 1: the detection of murine GFP+ MSCs within the full-thickness skin excision wounds in mice.
