# Supplementary material for: Novel Cryopreservation Approach Providing Off-the-Shelf Availability of Human Multipotent Mesenchymal Stromal Cells for Clinical Applications
Source: Stem Cells Int. 2019 Nov 22;2019:4150690. doi: 10.1155/2019/4150690 (PMC6907044; doi:10.1155/2019/4150690)
Supplement: Supplementary Materials — Figure S1: macroscopic images of wound closure. Figure S2: overall study design and achieved results. Supplementary file 1: the detection of murine GFP+ MSCs within the full-thickness skin excision wounds in mice. [file 4150690.f1.zip › 4150690.f1.pdf]

Supplementary

The detection of murine GFP+ MSCs within the full-thickness skin excision wounds in mice.

Materials and methods

Cell isolation and culture

Murine adipose tissue MSCs were isolated from GFP transgenic mice (FVB-Cg-Tg (GFPU) 5Nagy) and cultured as previously described for human MSCs.

Detection of GFP fluorescence in murine MSCs *in vitro* and *in vivo*

To evaluate the dependence of fluorescence intensity on cell viability of MSCs, part of the cells was lethally damaged by 3 repeated freeze-thaw cycles in the absence of cryoprotectant. The viability of cells was determined by trypan blue staining, the GFP fluorescence was detected by confocal microscopy Zeiss LSM 510 META (Carl Zeiss, Germany).

Prior to implantation, murine GFP+ MSCs were pre-treated with sucrose during 24 hrs and cryopreserved as previously described for human cells in PS1D medium. After thawing, cell suspension was mixed with 10% calcium chloride solution and blood serum at ratio 9:0.25:0.75. 50 μl of obtained mixture containing 0.5-0.3 × 10^6 cells were placed onto the wound surface.

The presence of cryopreserved murine GFP+ MSCs within full-thickness excision wounds was assessed after 30 min and then on day 1, 3 and 5 after implantation. Skin flaps from the wound area were dissected and examined without additional manipulations using a Zeiss LSM 510 META confocal microscope (Carl Zeiss, Germany).

Results

The detection of GFP fluorescence in intact and lethally damaged murine MSCs *in vitro*

For the accurate detection of GFP+ MSCs within the full-thickness wounds, the fluorescence of GFP has been studied *in vitro* in intact and lethally damaged cells (Fig. S3).

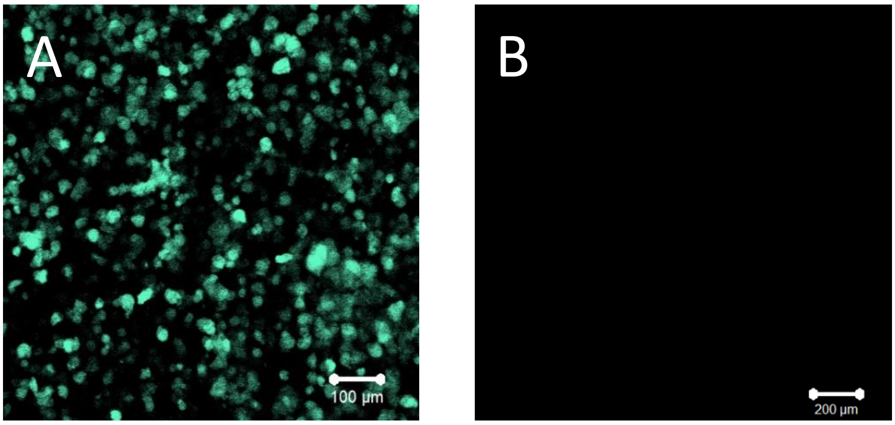

Fig. S3. The detection of GFP fluorescence in intact GFP+ MSCs (A) and lethally damaged GFP+ MSCs (B) *in vitro*.

After 3 repeated cycles of fast freezing and thawing, all MSCs were positively stained by trypan blue, confirming the cell membrane damage. Opposite to intact cells (Fig. S3A), no GFP fluorescence was detected in damaged MSCs (Fig. S3B). It was previously shown that the reduction of GFP fluorescence in adenocarcinoma 3230 cell line was strongly dependent on cell viability assessed by trypan blue or ethidium bromide staining [1].

The detection of cryopreserved GFP+ murine adipose tissue MSCs after implantation onto the full-thickness excision wounds in mice

After 30 min of implantation, GFP+ MSCs showed spherical morphology and distributed throughout the PS1D hydrogel (Fig. S4A). After 24 hrs, MSCs changed morphology to fibroblast-like (Fig. S4B). There were no visible signs of the viability reduction or decrease in cell number. However, following analysis showed the decrease in the number of GFP+ cells after 3 days (Fig. S4C) and almost complete absence of cells on day 5 post-implantation (Fig. S4D).

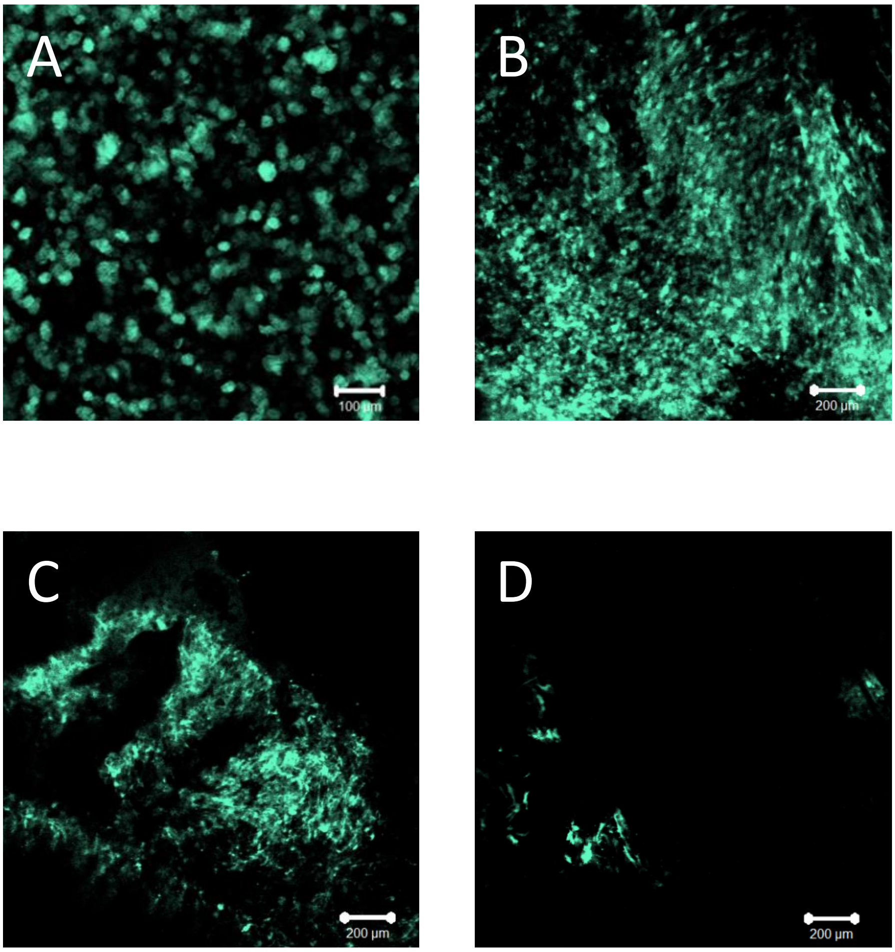

Fig. S4. The detection of cryopreserved GFP+ MSCs after implantation within the 3D PS1D hydrogel into the full-thickness excision wounds. A - 30 min after implantation, B – 1st day, C – 3rd day and D – 5th day (D) post-implantation.

These results confirm the retention of viable murine GFP+ MSCs up to 5 days after implantation into a full-thickness excision wounds in mice.

References

1. Elliott G, McGrath J, Crockett-Torabi E: **Green fluorescent protein: A novel viability assay for cryobiological applications.** *Cryobiology* 2000, **40:**360-369.
