# Supplementary figures and images for: Novel Cryopreservation Approach Providing Off-the-Shelf Availability of Human Multipotent Mesenchymal Stromal Cells for Clinical Applications
Source: Stem Cells Int. 2019 Nov 22;2019:4150690. doi: 10.1155/2019/4150690 (PMC6907044; doi:10.1155/2019/4150690)

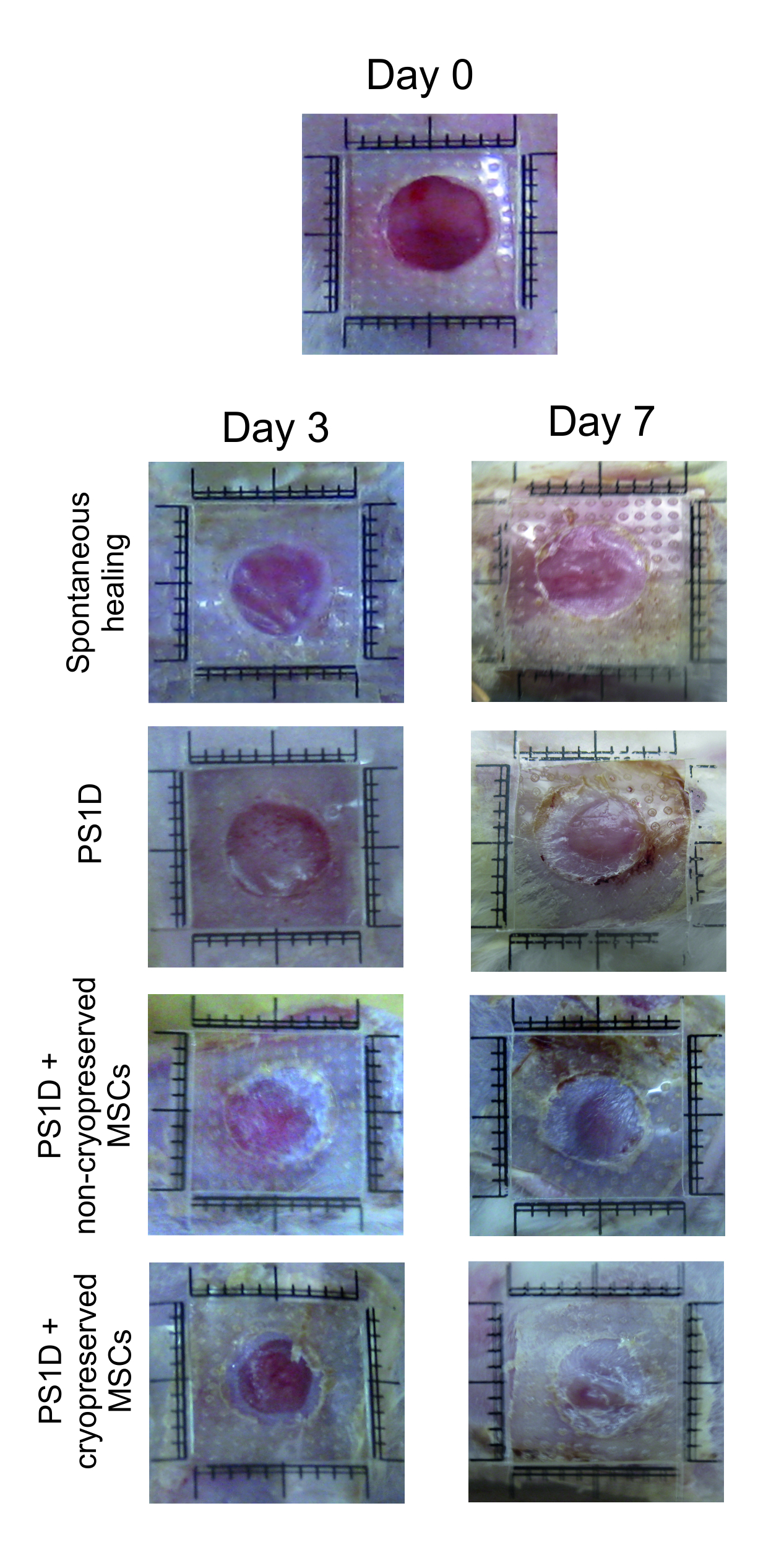

Supplement: Supplementary Materials — Figure S1: macroscopic images of wound closure. Figure S2: overall study design and achieved results. Supplementary file 1: the detection of murine GFP+ MSCs within the full-thickness skin excision wounds in mice. [file 4150690.f1.zip › Figure_S1.jpg]

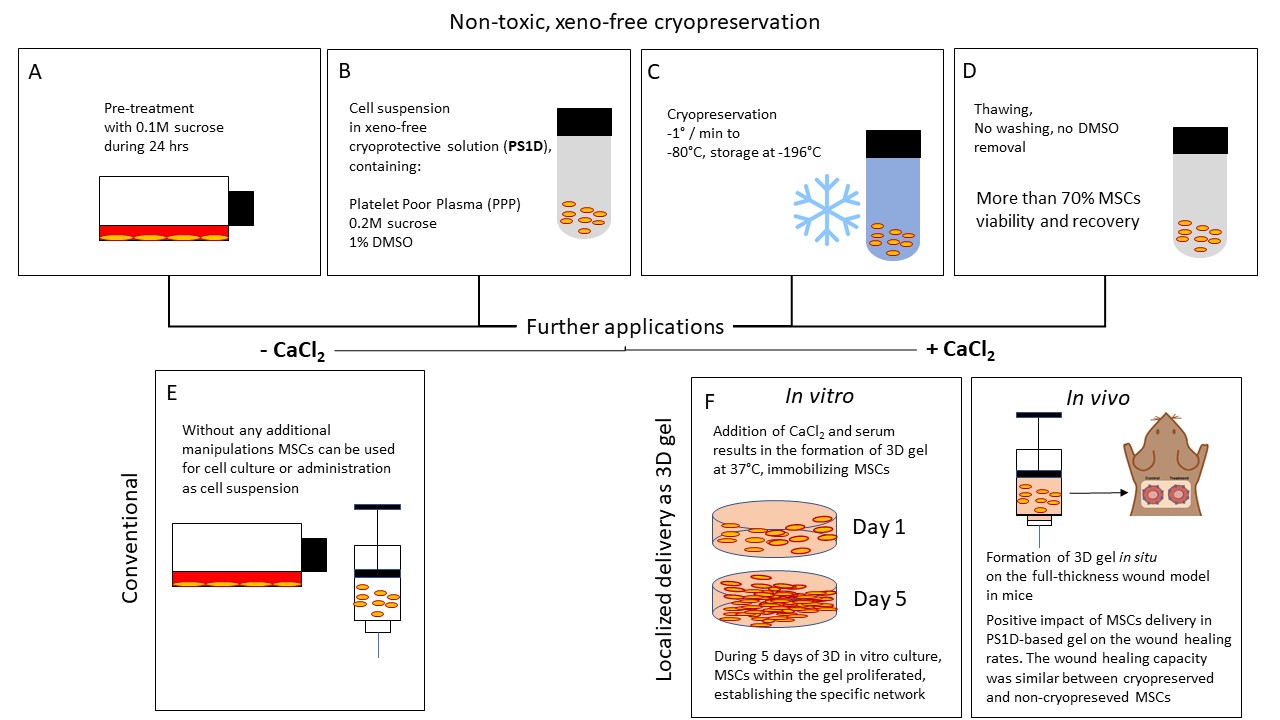

Supplement: Supplementary Materials — Figure S1: macroscopic images of wound closure. Figure S2: overall study design and achieved results. Supplementary file 1: the detection of murine GFP+ MSCs within the full-thickness skin excision wounds in mice. [file 4150690.f1.zip › Figure_S2.jpg]
